# Height-Related Polygenic Variants Are Associated with Metabolic Syndrome Risk and Interact with Energy Intake and a Rice-Main Diet to Influence Height in KoGES

**DOI:** 10.3390/nu15071764

**Published:** 2023-04-04

**Authors:** Sunmin Park

**Affiliations:** Department of Food and Nutrition, Obesity/Diabetes Research Center, Hoseo University, 165 Sechul-Ri, BaeBang-Yup, Asan-Si 336-795, ChungNam-Do, Republic of Korea; smpark@hoseo.edu; Tel.: +82-41-540-5633; Fax: +82-41-540-5638

**Keywords:** stature, *GDF5*, *IGF-1R*, polygenic risk score, in silico analysis, energy intake

## Abstract

Adult height is inversely related to metabolic syndrome (MetS) risk, but its genetic impacts have not been revealed. The present study aimed to examine the hypothesis that adult height-related genetic variants interact with lifestyle to influence adult height and are associated with MetS risk in adults aged >40 in Korea during 2010–2014. Participants were divided into short stature (SS; control) and tall stature (TS; case) by the 85th percentile of adult height. The genetic variants linked to adult height were screened from a genome-wide association study in a city hospital-based cohort (n = 58,701) and confirmed in Ansan/Ansung plus rural cohorts (n = 13,783) among the Korean Genome and Epidemiology Study. Genetic variants that interacted with each other were identified using the generalized multifactor dimensionality reduction (GMDR) analysis. The interaction between the polygenic risk score (PRS) of the selected genetic variants and lifestyles was examined. Adult height was inversely associated with MetS, cardiovascular diseases, and liver function. The PRS, including zinc finger and BTB domain containing 38 (*ZBTB38*)_rs6762722, polyadenylate-binding protein-interacting protein-2B (*PAIP2B*)_rs13034890, carboxypeptidase Z (*CPZ*)_rs3756173, and latent-transforming growth factor beta-binding protein-1 (*LTBP1*)_rs4630744, was positively associated with height by 1.29 times and inversely with MetS by 0.894 times after adjusting for covariates. In expression quantitative trait loci, the gene expression of growth/differentiation factor-5 (*GDF5*)_rs224331, non-SMC condensin I complex subunit G (*NCAPG*)_rs2074974, ligand-dependent nuclear receptor corepressor like (*LCORL*)_rs7700107, and insulin-like growth factor-1 receptor (*IGF1R*)_rs2871865 was inversely linked to their risk allele in the tibial nerve and brain. The gene expression of *PAIP2B*_rs13034890 and a disintegrin and metalloproteinase with thrombospondin motifs-like-3 (*ADAMTSL3*)_rs13034890 was positively related to it. The PRS was inversely associated with MetS, hyperglycemia, HbA1c, and white blood cell counts. The wild type of *GDF5*_rs224331 (Ala276) lowered binding energy with rugosin A, D, and E (one of the hydrolyzable tannins) but not the mutated one (276Ser) in the in-silico analysis. The PRS interacted with energy intake and rice-main diet; PRS impact was higher in the high energy intake and the low rice-main diet. In conclusion, the PRS for adult height interacted with energy intake and diet patterns to modulate height and was linked to height and MetS by modulating their expression in the tibial nerve and brain.

## 1. Introduction

Adult height is achieved by growth during childhood and adolescence, with full height attained at age 18–20. The adult height of a child or adolescent between the age of 4 to 17.5 years can be predicted based on the current height, body mass, chronological age, and parents’ height. The genetic impact on height increases can be estimated from adult height. However, after 30 years of age, people, especially women, gradually lose height. A loss of height of about 2.54 cm for men and 5.08 cm for women occurs between ages 30 to 70 years. However, a remarkable decline in height indicates certain health problems. Loss of height with age may occur due to poor nutrition, compression, and dehydration of the discs between the vertebrae, curvature of the spine, low bone mineral density, diabetes, and low muscle mass in the torso [1]. Therefore, when adjusted for osteoporosis, skeletal muscle mass, nutritional status, and age, the current adult height would reflect height in the 20s [2]. The genetic variants associated with adult height can be analyzed through genome-wide association studies (GWAS) after adjusting for age, gender, osteoporosis, income, body mass index (BMI), education, and energy intake. The interaction between genetics and lifestyle factors can be evaluated. 

Human height is modulated by genetic factors that determine about 80 percent of an individual’s height, and it is a polygenic trait. The GWAS of adult height has shown that common variants account for 50% of height variations [3,4]. However, the influencing genetic variants are only partially understood. Studies determining genetic variants associated with height have been conducted in not only children but also adults. The genetic variants related to adult height were identified as solute carrier family-27 (*SLC27A3*) and cytochrome P450, family 26, subfamily B (*CYP26B1*) in Japan [5], and insulin-like growth factor (*IGF)-2/H19* and Tet methylcytosine dioxygenase-1 (*TET1*) in Icelanders [6]. In a study on 8842 Koreans, several adult height-related loci were identified, such as exostosin glycosyltransferase-1 (*EXT1*), FRAS1-related extracellular matrix-1 *(FREM1*), paralemmin-2 (*PALM2*), A-kinase anchor protein-2 (*AKAP2*), nucleoporin-37 *(NUP37)*, pro-melanin concentrating hormone (*PMCH*), *IGF1*, keratin-20 (*KRT20*), and ankyrin repeat domain-60 (*ANKRD60*). Their risk alleles explain about 1.0% of the height variations among Koreans [7]. In a pediatric cohort with European ancestry, 16 genetic variants in epidermal growth factor containing fibulin extracellular matrix protein-1 (*EFEMP1*)-polyribonucleotide nucleotidyltransferase-1 (*PNPT1*), G protein-coupled receptor-126 (*GPR126*), *C6orf173*, sperm associated antigen-17 (*SPAG17*), *histone class-1*, human leukocyte antigen (*HLA) class III*, and growth differentiation factor-5 (*GDF5*)-ubiquinol-cytochrome c reductase complex chaperone CBP3 homolog (*UQCC*) were associated with height, which explains 1.64% of the variations in height [8]. In Chinese children, zinc finger and BTB domain containing 38 (*ZBTB38*), zinc finger protein 638 (*ZNF638*), ligand-dependent nuclear receptor corepressor like (*LCORL*), cyclin-dependent kinase10 (*CDK10*), *Cdk5 and Abl enzyme substrate 1* (*CABLES1*), and tRNA-splicing endonuclease subunit-15 (*TSEN15*) related genetic variants were revealed to be linked to height [9]. 

Adult height is demonstrated to be associated with several metabolic and other diseases. In one study, adult height is found to have an inverse relationship with 2-h serum glucose concentrations only in adults with <35 kg/m^2^ BMI during oral glucose tolerance tests [10], suggesting the height effects can be offset with adiposity to influence blood glucose levels. Furthermore, the genetic variants related to height have shown positive and inverse associations with various metabolic diseases, such as atrial fibrillation (OR = 1.33, 95% CI = 1.26–1.40) [11], venous thromboembolism (OR = 1.15, 95% CI = 1.11–1.19), hip fracture (OR = 1.27, 95% CI = 1.17–1.39), hypertension (OR = 0.88, 95% CI = 0.85–0.91), and coronary artery disease (OR = 0.86, 95% CI = 0.82–0.90) [12]. Multiple height-associated pathways link height with various diseases indicating that multiple biological mechanisms affecting height raise the risk of these diseases [12]. Therefore, height and metabolism may be linked to height-related genetic variants. 

Few studies have examined the association between height-related genetic variants and metabolic syndrome (MetS). It was hypothesized that the genetic variants related to adult height were associated with MetS risk and that they interacted with lifestyle factors. The purpose of the current study was to examine the hypothesis by (1) exploring the genetic variants linked to tall stature after adjusting for the factors associated with a reduction in height among adults, (2) generating the optimal genetic model for tall stature, (3) analyzing the association of polygenic risk scores (PRS) with the risk of MetS and its components, and (4) examining the interaction of PRS with lifestyle factors to affect adult height in the city hospital-based cohort.

## 2. Methods and Materials

### 2.1. Participants

Adult volunteers aged over 40 years were recruited in a city-hospital cohort (n = 58,701), the Ansan/Ansung cohort (n = 5493), and a rural cohort (n = 8105) in the Korean Genome and Epidemiology Study (KoGES) during the period 2010–2014 [13]. The objectives were primarily evaluated in the city-hospital cohort in the present study, and the Ansan/Ansung plus rural cohorts were applied as replicate studies for GWAS results for height. The institutional review board (IRB) of the Korea National Institute of Health approved the KoGES (KBP-2015-055), and the IRB of Hoseo University approved the present study (HR-034-01). All participants signed a written informed consent form. 

### 2.2. Adult Height Criteria

The short stature (SS; n = 51,165; control) and tall stature (TS; n = 7536; case) groups of the participants were divided by the cutoff of ≥175 cm for men and ≥163 cm for women as tall stature, and below that for short stature. These heights were the 85th percentiles of adult height for people aged >40 between 2010–2014 in Korea.

### 2.3. Survey Questionnaires and Anthropometric and Biochemical Measurements 

On their first visit, the participants completed the demographic questionnaires. Height and weight were measured with an automatic digital machine wearing light gowns and bare feet. The appendicular skeletal muscle mass in the city hospital cohort was predicted using a prediction model generated by the XGBoost algorithm in the Ansan/Ansung cohort [14]. Blood pressure was determined with a sphygmomanometer in a sitting position three times, and average systolic (SBP) and diastolic blood pressure (DBP) were recorded. The participants fasted for over 12 h. The serum and plasma were collected from the blood drawn without and with ethylenediaminetetraacetic acid (EDTA) and heparin, respectively. Biochemical parameters in the serum were measured with previously described standard methods [15]. They included glucose, triglyceride, total cholesterol, HDL, alanine aminotransferase (ALT), aspartate aminotransferase (AST), and creatinine concentrations in the serum, and blood hemoglobin A1c (HbA1c) and white blood cell (WBC) contents. The estimated glomerular filtration rate (eGFR) was calculated using the equation generated by the Modification of Diet in Renal Disease study [16]. The insulin resistance was calculated using the prediction model generated by the homeostatic model assessment of insulin resistance (HOMA-IR) in the Ansan/Ansung cohort from a previous study [17].

Details of lifestyle factors, including food intake, were collected using questionnaires during a health interview by a trained technician. Daily alcohol and coffee intakes were calculated by multiplying their intake amounts with frequencies. Smoking status was classified into non-, past, and current smokers. Smokers were specified as smoking >100 cigarettes in their lifetime, and current smokers who had not smoked for 6 months were considered past smokers. Exercise status was classified into regular exercise and no exercise based on the cutoff of 30 min of moderate-intensity activity such as water pushing a lawn mower, brisk walking, hiking, aerobics, riding a bike, dancing, doubles tennis, or rollerblading for ≥3 days per week.

### 2.4. Usual Food Intake Measurement

The usual food and nutrient consumption during the last 12 months was determined based on the response to a semi-quantitative food frequency questionnaire (SQFFQ) generated for Koreans. It included 106 foods Koreans commonly consumed. The SQFFQ was confirmed with three-day food records for the four seasons in a previous study [17]. The food frequencies were divided into 8 categories, such as seldom, once per month, two to three times per month, once or twice per week, three or four times per week, five or six times per week, daily, twice a day, and ≥3 times a day. One portion size of each food was classified into either more than, equal to, or half of the portion size shown in a photograph. Participants selected the frequencies and one portion size of each food item. Their intake was calculated by multiplying the median of the frequencies by one portion size of each food. Daily nutrient intake was estimated from the calculated daily food amount using the computer-aided nutritional analysis program CAN-Pro 2.0 (Korean Nutrition Society, Seoul, Republic of Korea). Daily nutrient intake was calculated by summing the individual nutrient of each food. 

### 2.5. Dietary Patterns and Dietary Inflammatory Index (DII)

The dietary patterns were clustered with principal component analysis (PCA) of 30 predefined groups categorized from the 106 food items. Four dietary patterns were designated based on eigenvalues >1.5, orthogonal rotation procedure (varimax), and ≥0.40 factor-loading values. The four dietary patterns were named as Korean-balanced diet (KBD), Western-style diet (WSD), plant-based diet (PBD), and rice-main diet (RMD), according to foods. The dietary inflammation index (DII) was calculated with a prediction equation generated with the food and nutrient intake and divided by 100 [18]. 

### 2.6. Genotyping, Its Quality Control, and Genotype-Tissue Expression (GTEx)

Genomic DNA was isolated from the volunteers’ blood and genotyping was performed using a Korean Chip (Affymetrix, Santa Clara, CA, USA). It was made to evaluate the genetic impact of single nucleotide polymorphisms (SNPs) on metabolic diseases in Koreans at the Center for Genome Science at the Korea National Institute of Health [19]. The genotypes were imputed based on the Korean Haplotype Map (HapMap) data [20]. The genotyping accuracy was assessed with a Bayesian learning algorithm for Robust General Linear Models (RGLMs) [21]. Genotyping accuracy was checked with the exclusion criteria as follows: <98% genotyping accuracy, ≥30% heterozygosity, ≥4% missing genotype call rate, ≤1% minor allele frequency (MAF), and *p* ≤ 0.05 Hardy–Weinberg equilibrium (HWE) [21]. The significance distribution of the genetic variants was visualized with a Manhattan plot by the R program. A Q–Q probability plot indicates the goodness of fit of the actual data distribution to the theoretical data distribution. When the lambda value in the Q–Q plot was close to 1, the genotypes were ideal. Among the selected genotypes, the pathway associated with height was selected with satisfying the P value for Bonferroni correction using the multi-marker analysis of genomic annotation (MAGMA) gene-set analysis in the SNP2GENE function of the functional mapping and annotation (FUMA) web application (https://github.com/Kyoko-wtnb/FUMA-webapp/, accessed on 20 April 2022).

Genotype-Tissue Expression (GTEx) was used to show the gene expression with different genetic variant alleles in various tissues using the GTEx expression quantitative trait loci (eQTL) calculator (https://gtexportal.org/home/testyourown, accessed on 3 May 2022). 

### 2.7. Selection of Genetic Variants to Influence Adult Height and Their Optimal Model with the SNP–SNP Interaction

**Figure 1:**
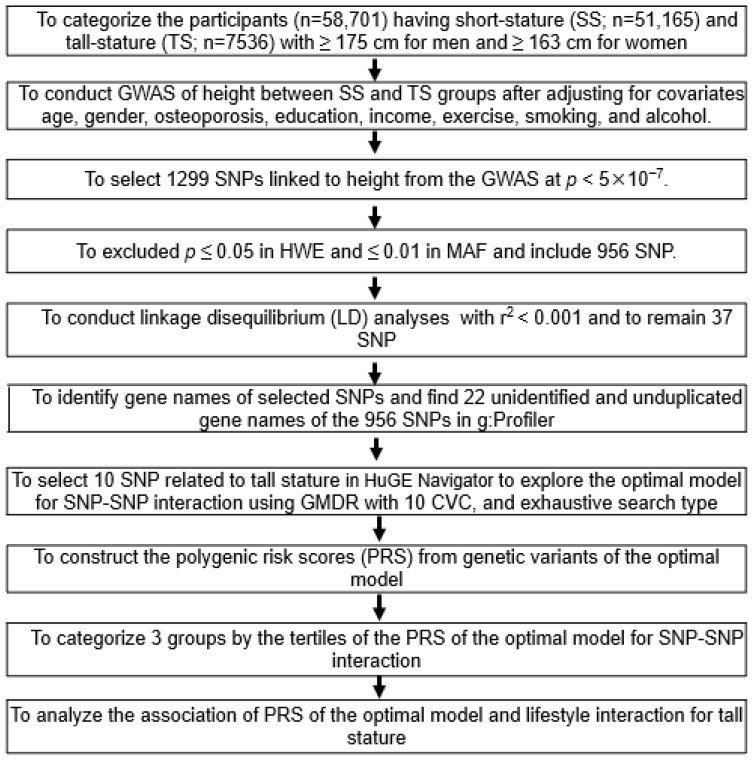
Flow chart to generate polygenic risk score (PRS) associated with stature by SNP–SNP interaction and its interaction with lifestyle factors. The short stature (SS; n = 51,165; control) and tall stature (TS; n = 7536; case) groups of the participants were divided by the cutoff of ≥175 cm for men and ≥163 cm for women, which were the 15th percentiles of adult height in people aged >40 years in 2010–2014 in Korea.

Figure 1 provides the optimal genetic model selection process. First, genetic variants (n = 1299) influencing height were selected using the SS and TS groups in the city hospital-based cohort at a significance level of *p* < 5 × 10^−7^, as defined above. Of these, 956 genetic variants satisfied MAF (>1%) and HWE (*p* > 0.05), and the 79 unduplicated gene names were identified using g:Profiler (https://biit.cs.ut.ee/gprofiler/snpense, accessed on 7 March 2022). The genetic variants with high linkage disequilibrium (LD; D′ ≥ 0.2) were excluded due to giving the same genetic impact on the height. After exclusions, 37 genetic variants remained to meet the criteria (D′ < 0.2) using Haploview 4.2 in the PLINK toolset, and 15 with the same or unidentified gene names were eliminated. 

Ten SNPs were included in the optimal genetic model by selecting them based on their interaction with each other from 22 genetic variants in the generalized multifactor dimensionality reduction (GMDR) software version 2.0 using the exhaustive search type after adjusting for covariates. Covariate set 1 contained age, gender, residence area, education, and income, and covariate set 2 included covariates in set 1 plus energy intake, alcohol intake, regular exercise, and smoking status for model 2. The selection criteria were a *p* < 0.001 for the sign test of the testing balanced accuracy (TEBA) and ten cross-validation consistency (CVC) in the ten-fold cross-validation [22]. Among the optimal models to satisfy the significance level of the sign test and CVC, the model with the lowest number of genetic variants and highest odds ratios (ORs) was selected as the optimal model. 

The PRS for the optimal genetic model was calculated as a sum of the number of the risk alleles from each selected SNP in the optimal SNP–SNP interaction model [23,24,25]. As the SNP risk allele was G, the genetic scores of “AA”, “AG”, and “GG” were 0, 1, and 2, respectively. Each model was divided into Low-, Middle-, and High-PRS. The PRS in the four-SNP model was classified into Low-, Middle-, and High-PRS based on scores of 0–3, 4–5, and ≥6, respectively; and that in the seven-SNP model was categorized into 2–7, 8–10, and ≥11, respectively. The PRS of the model was further used for analyzing the interaction with the lifestyle parameters.

### 2.8. Molecular Docking of Wild and Mutated GDF5 with Food Compounds

The wild and mutated chemical structures of the proteins *GDF5*_rs224331 (Ala276Ser) were made in the Protein Data Bank (PDB) format from the Iterative Threading Assembly Refinement (I-TASSER) website (https://zhanggroup.org/I-TASSER/ (accessed on 20 June 2022). Food compounds (n = 20,000) were downloaded from the fooDB website (https://foodb.ca/, accessed on 9 June 2022). Water molecules attached to the ligands were eliminated using the pleomorphic analysis methodology (PyMOL) software version 2.0 (DeLano Scientific LLC, South San Francisco, CA, USA) [26]. The PDB structure of the protein and ligand was changed into a protein data bank, partial charge (Q), and atom type (T) (PDBQT) files lattice format using AutoDock Tools 1.5.6 (Molecular Graphics Laboratory, Scripps Research Institute, La Jolla, CA, USA) [26]. The active sites of the proteins GDF5 were identified using the ProteinsPlus website (https://proteins.plus/, accessed on 8 July 2022), and they and the mutated sites (rs224331) were included for molecular docking. 

Food components with a <−10 kcal/mol binding free energy were selected [27]. The binding free energy at the active site indicated the binding affinity of *GDF5* to the food compound. The lower the binding free energy, the tighter the binding and affinity.

### 2.9. Molecular Dynamics Simulation (MDS)

The conformational changes in the GDF5 structures were examined using MDS to detect the changes in their activity. After the top docking poses with the selected food compounds were inserted, simulations were conducted on the *GDF5* active site and docked complexes. Additionally, the Chemistry at Harvard Macromolecular Mechanics (CHARMM) force field was added to each molecular structure generated by “Simulation”, and the protein was solvated by “Solvation”. The “Standard Dynamics Cascade” was used to set the molecular dynamics simulation parameters for the protein added to the solvent system. The ramp-up time, equilibration time, simulation sampling time, and simulation step size were set to 40 ps, 400 ps, 10,000 ps, and 2 fs, respectively, and other parameters were set to default values. The hydrogen bond values, root mean square deviation (RMSD), and root mean square fluctuations (RMSF) were analyzed after the 10 ns simulation.

### 2.10. Statistical Analysis 

The sample size (n = 58,701) was sufficient to show significance at α = 0.05 and β = 0.99 when assigned an odds ratio (OR) of 1.08 in the logistic regression analysis using a G-power calculator. Descriptive statistical analysis was conducted using SAS (version 9.3; SAS Institute, Cary, NC, USA). Frequency distributions were used for the categorical variables, and their statistical differences between the SS and TS groups were calculated with the Chi-square test. The adjusted means and standard deviations for the continuous variables were assessed after adjusting the covariates, including age, gender, education, income, survey year, BMI, osteoporosis, energy intake, exercise, alcohol intake, and smoking status. The statistical differences between the genders and adult height groups were analyzed using a two-way analysis of covariance (ANCOVA) with adjustment for the covariates [28]. When ANCOVA was significant, multiple comparisons according to the genders and height groups were performed using Tukey’s test. 

The association of height with anthropometric and biochemical parameters was analyzed using logistic regression analysis after adjustment for covariates. The results are provided with each metabolic parameter’s adjusted odds ratios (ORs) and 95% confidence intervals (CI). Two different covariate sets were included according to the covariates. Covariate set 1 included age, place of residence, survey year, osteoporosis, BMI, education, and income as covariates. Covariate set 2 was calculated with covariates of model 1 plus energy intake, physical activity, smoking status, and alcohol intake. 

The two-way ANCOVA was conducted with the main effect terms of PRS and lifestyles, their interaction term, and covariates. If the interaction terms showed statistical significance, each lifestyle parameter was categorized into high or low groups according to the dietary reference intake [29] or their 30th percentile. The ORs and 95% CI of the height with PRS were also assessed with logistic regression analysis in each of the high or low groups in the lifestyle-related parameters. Any significant difference in the height was determined according to the PRS groups using the χ^2^ test or ANCOVA in the lifestyle-related parameters’ low- or high-groups. 

## 3. Results

### 3.1. Demographic Characteristics and Lifestyles According to Gender and Adult Height

The age was higher in the SS group than in the TS group in both genders (Table 1). The proportion of tall persons was higher than that of short persons in men, and it was the opposite in women. The proportion of both men and women in the TS who lacked higher education (≤middle school) was much lower than in the SS group (Table 1). However, the proportion of men with a high income was lower in the TS than in the SS group, but it was the reverse in women. Smoking status was not significantly different between the SS and TS groups in both genders (Table 1). The proportion of the participants with regular physical exercise was small but significantly higher in the TS group than in the SS group, but only in women. Only in men, alcohol intake was higher in the TS group than in the SS group (Table 1). 

### 3.2. Nutrient Intake According to Gender and Adult Height

Energy intake was lower in the SS than in the TS group in both genders. Regarding the intake of macronutrients, fat intake was lower in the SS group than in the TS group only in women, and the trend of carbohydrate intake was the opposite of that of the fat intake (Table 1). However, protein intake was not significantly different between the SS and TS groups for both genders. Dietary fiber intake was lower in the SS group than in the TS group only in men, and calcium and vitamin C and D intakes were lower in the SS group than in the TS group for both genders (Table 1). Intakes of DII and flavonoids did not differ between the TS and SS groups (Table 1). The proportion of KBD was lower in the SS group than in the TS group only in men, but that of PBD and RMD was lower in the SS group than in the TS group only in women (Table 1). The proportion of WSD was much lower in the SS group than in the TS group in both genders. Coffee intake, but not tea intake, was lower in the SS group than in the TS group in women (Table 1). 

### 3.3. Prevalence of MetS and Its Related Parameters According to Gender and Adult Height

As expected, height was much greater in the TS group than in the SS group in both genders. When the increase in height ceased at age 18, the weight was not significantly different between the TS and SS groups (Table 2). In women, the BMI during the study was higher in the SS group than the TS group and was inversely associated with height (adjusted ORs = 0.908). Waist circumferences were lower in the TS group than in the SS group for both genders. The inverse association of waist circumference with height was much more significant than the association with BMI (adjusted ORs = 0.327) (Table 2). The skeletal muscle index (SMI) and fat mass were significantly lower in the TS group than the SS group in both genders and was inversely associated with height. White blood corpuscles (WBC), which reflect the immune status, showed a trend similar to the SMI and fat mass (Table 2). However, there was no significant difference in serum high-sensitive C-reactive protein (hs-CRP) concentrations, an inflammatory marker, between the TS and SS groups (Table 2). 

MetS and cardiovascular disease (CVD) incidence was inversely associated with height. Fasting serum glucose concentrations and blood HbA1c levels were higher in the SS group than in the TS group for both genders, and they showed an inverse relationship with height (Table 2). Insulin resistance also showed an inverse association with height (Table 2). Dyslipidemia was also inversely related to height, showing that short persons had hyper-total cholesterol, hypo-high-density lipoprotein (HDL), hyper-low-density lipoprotein (LDL), and hyper-triglycerides in the blood, compared to tall persons (Table 2). SBP and DBP were higher in the SS group than in the TS group and revealed an inverse association with height (Table 2). Serum alanine aminotransferase (ALT) and aspartate aminotransferase (AST), the indexes of liver damage, had an inverse association with height. However, the eGFR estimated with serum creatinine concentrations was not significantly linked to height (Table 2). Furthermore, arthritis incidence, but not osteoporosis, was inversely associated with height (Table 2). 

### 3.4. Genetic Variants Linked to Adult Height

The genetic variants affecting adult height are shown as a Manhattan plot in Supplementary Figure S1A, and those satisfying the selection criteria were located in chromosomes 2, 3, 5, 6, 12, 15, and 20. The Q–Q plot in Supplementary Figure S1B indicated that the genetic variants did not deviate from the expected values (lambda = 1.032). 

The genetic variants that interacted with each other to promote an increase in height are presented in Table 3. Polyadenylate-binding protein-interacting protein 2B (*PAIP2B*)_rs13034890, carboxypeptidase Z (CPZ)_rs3756173, non-SMC condensin I complex subunit G (*NCAPG*)_rs2074974, *LCORL*_rs7700107, a disintegrin and metalloproteinase with thrombospondin motifs-like-3 (*ADAMTSL3*)_rs1600640, and insulin-like growth factor-1 receptor (*IGF1R)*_rs2871865 had an inverse association with height whereas latent-transforming growth factor beta-binding protein-1 (*LTBP1*)_rs4630744, DIS3-like exonuclease 2 (*DIS3L2*)_rs1249260, *ZBTB38*_rs6762722, and *GDF5*_rs224331 were positively associated with height (Table 3). The adjusted ORs of the genetic variants were between 0.8078 and 1.191, indicating a small effect, although each showed a highly significant *p*-value (5 × 10^−7^) in the city hospital-based cohort. However, their significance level in the Ansan/Ansung plus rural cohorts was 0.02–6.8 × 10^−5^. *GDF5*_rs224331 was located in the exon and was a missense mutation. These results indicated that the selected genetic variants were weakly associated with adult height (Table 3).

### 3.5. SNP–SNP Interaction by GMDR

**Figure 2:**
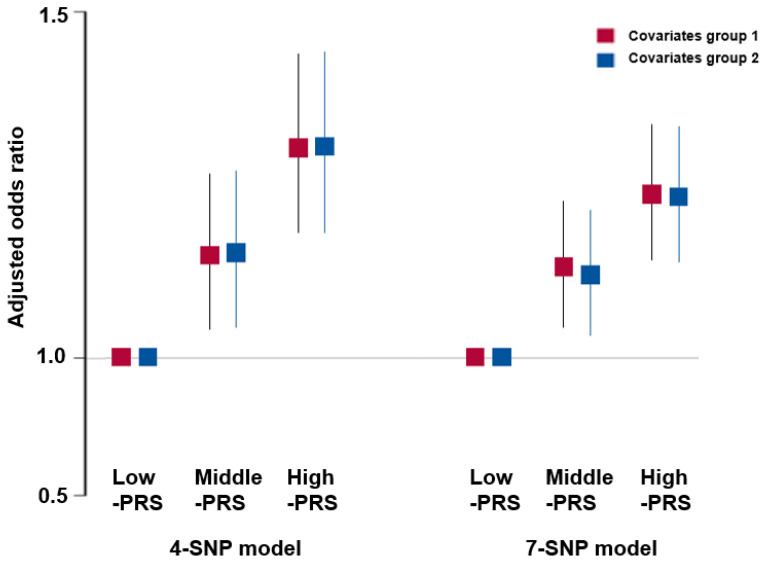
Adjusted odds ratio (ORs) and 95% confidence intervals (CI) of 4-SNP PRS and 7-SNP PRS models for tall stature PRS was generated with the sum of the number of risk alleles in each SNP, and it was classified as Low-PRS, Middle-PRS, and High-PRS according to the range 0–3, 4–5, and ≥6 in the four-SNP model and 0–5, 6–7, and ≥8 in the six-SNP model, respectively. Covariates of group 1 included age, gender, weight, residence area, education, and income, and those of group 2 contained those in group 1 plus energy intake, exercise, alcohol drinking, smoking, and osteoporosis incidence.

The genetic models, including 4, 7, 8, 9, and 10 genetic variants, met the selection criteria for the optimal model. The four-SNP model included *ZBTB38*_rs6762722, *PAIP2B*_rs13034890, *CPZ*_rs3756173, and *LTBP1*_rs4630744 whereas the seven SNP model contained *ZBTB38*_rs6762722, *PAIP2B*_rs13034890, *GDF5*_rs143384, *LCORL*_rs7700107, *DIS3L2*_rs1249260, *LTBP1*_rs4630744, and *NCAPG*_rs2074974 (Supplementary Table S1). Height was 1.29 times and 1.22 times positively associated with PRS for models 4 and 7, respectively, after adjusting for covariate groups (group 1 included age, sex, weight, education, income, and place of residence; group 2 contained covariate group 1 plus energy intake, alcohol intake, physical activity, and smoking, as shown in Figure 2). Therefore, the adjusted OR of the PRS was higher than that of the single SNP model. However, the ORs of the 4-SNP and 7-SNP models were similar, and the PRS of the 4-SNP model was appropriate. The four-SNP model was, therefore, more appropriate to explain the genetic impact on height. 

### 3.6. Expression of Quantitative Trait Loci (eQTL) of the Selected Genes According to the Alleles

**Figure 3:**
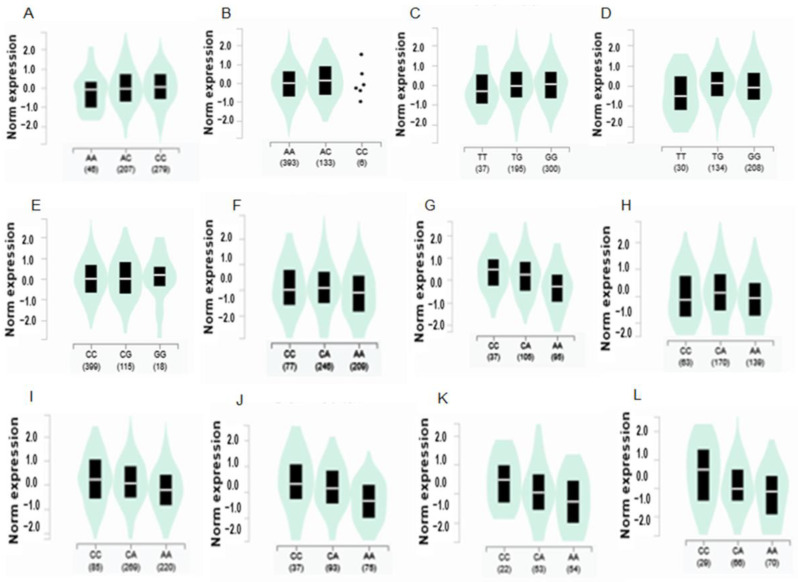
Gene expression according to the alleles of the selected SNPs for high body fat risk in different tissues. The black box indicated the box plot of norm expression of the gene according to each allele. The middle line was the median of the norm expression of gene with each allele. The green bell shape showed the distribution of norm gene expression with each allele. (**A**) *NCAPG*_rs2074974 in the tibial nerve (β = 0.14; *p* = 0.003). (**B**) *LCORL*_rs7700107 in the tibial nerve (β = 0.091; *p* = 0.0092). (**C**) *ADAMTSL3*_rs1600640 in the tibial tissue (β = 0.12, *p* = 0.0015). (**D**) *ADAMTSL3*_rs1600640 in the atrial appendage of the heart (β = 0.16, *p* = 2.1 × 10^−7^). (**E**) *IGF-1R*_rs2871865 in the tibial tissue (β = 0.11, *p* = 0.011). (**F**) *GDF5*_rs224331 in the tibial tissue (β = −0.13, *p* = 0.0095). (**G**) *GDF5*_rs224331 in the pituitary (β = −0.42, *p* = 4.6 × 10^−12^). (**H**) *GDF5*_rs224331 in the atrial appendage of the heart (β = −0.14, *p* = 0.0071). (**I**) *GDF5*_rs224331 in the thyroid (β = −0.25, *p* = 1.1 × 10^−8^). (**J**) *GDF5*_rs224331 in the cortex of the brain (β = −0.41, *p* = 8.2 × 10^−6^). (**K**) *GDF5*_rs224331 in the hippocampus of the brain (β = −0.38, *p* = 0.000049). (**L**) *GDF5*_rs224331 in the amygdala of the brain (β = −0.33, *p* = 0.003).

The genetic expression of the variant alleles of *GDF5, NCAPG, LCORL, PAIP2B, ADAMTSL3*, and *IGF1R* was different across different tissues. The tibial nerve showed the maximum differences in the gene expression of the alleles. The gene expression of *NCAPG*_rs2074974 decreased with the risk allele (A) compared to the non-risk allele in the tibial nerve (slope = 0.14, *p* = 0.003), and *IGFR1* also showed a similar trend in the tibial nerve (slope = 0.11, *p* = 0.011) (Figure 3). However, *ADAMTSL3*_rs1600640 showed an increased expression with the risk allele compared to the non-risk allele in the tibial nerve (slope = 0.16, *p* = 0.0015) and the arterial appendage of the heart (slope = 0.16, *p* = 2.1 × 10^−7^). The risk allele of *PAIP2B*_rs13034890 had a lower expression than the non-risk allele in the pituitary (slope = −0.29, *p* = 0.000083). Interestingly, the expression of *GFD5*_rs224331 showed a significant decrement in the risk allele compared to the non-risk allele in various tissues, especially the cortex, hippocampus, and amygdala of the brain, tibial nerve, thyroid, and heart (slope = −0.12~−0.41, *p* = 0.0095–1.1 × 10^−8^) (Figure 3). These results indicated that the gene expressions in the tibial nerve could act as central modulators of height and that the selected genetic variants in the tibial nerve play a critical role in height. 

### 3.7. Binding Affinity of Hydrolyzable Tannins to GDF5_rs224331

**Figure 4:**
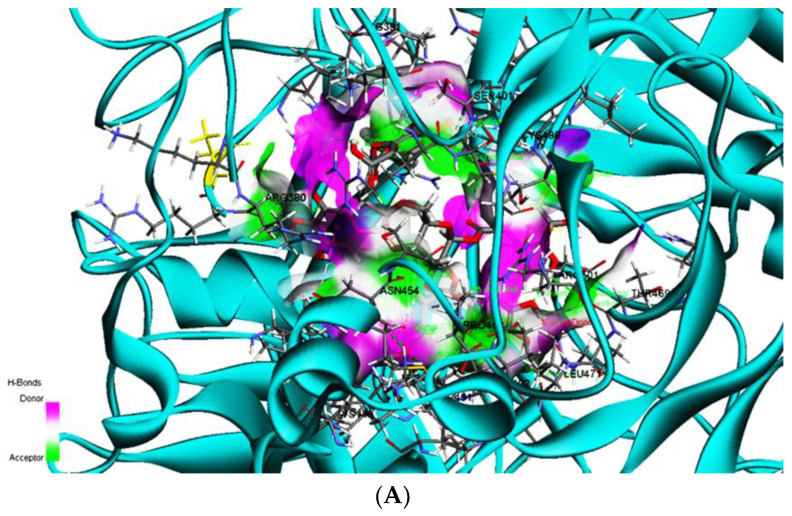

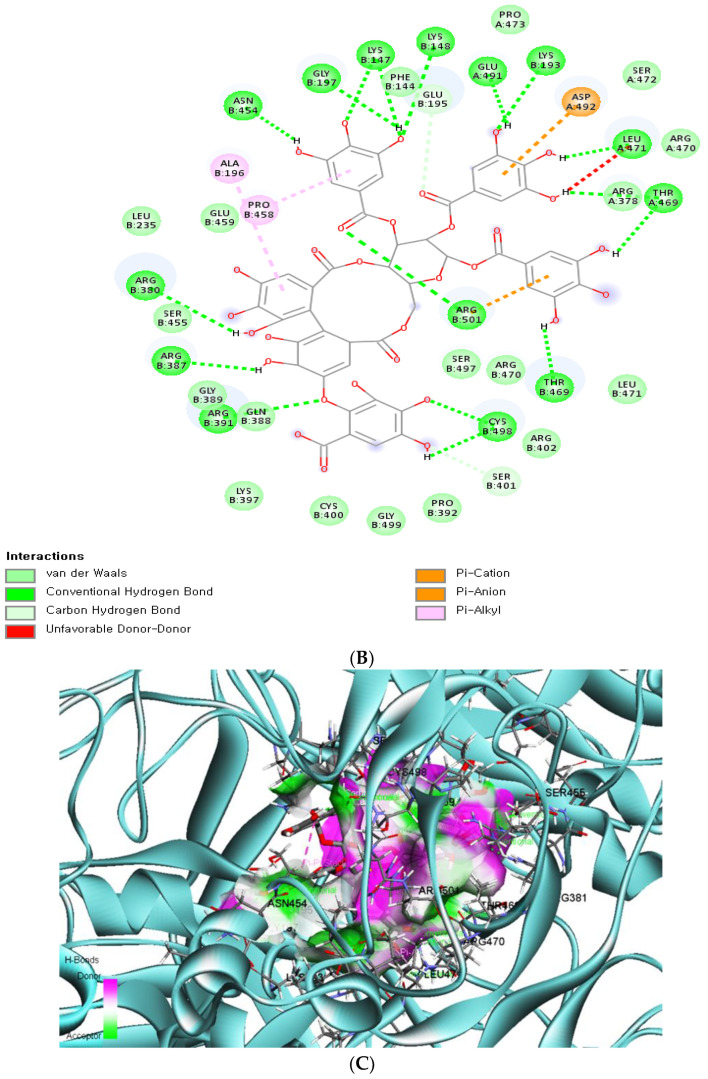

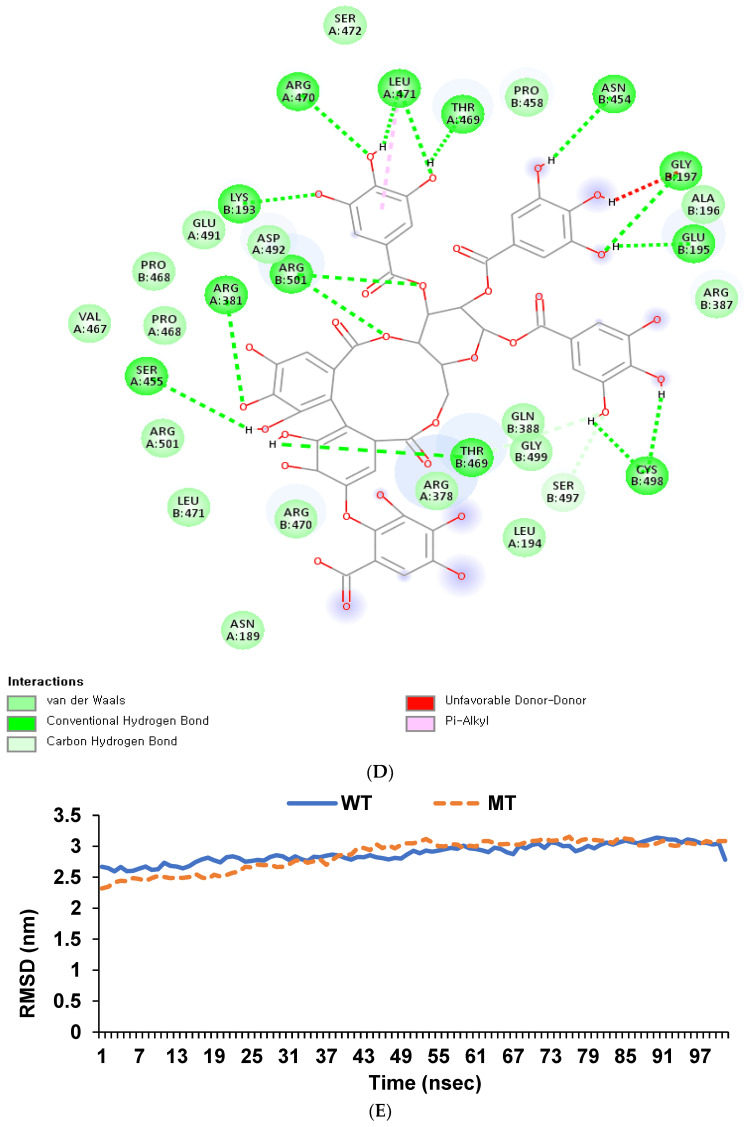

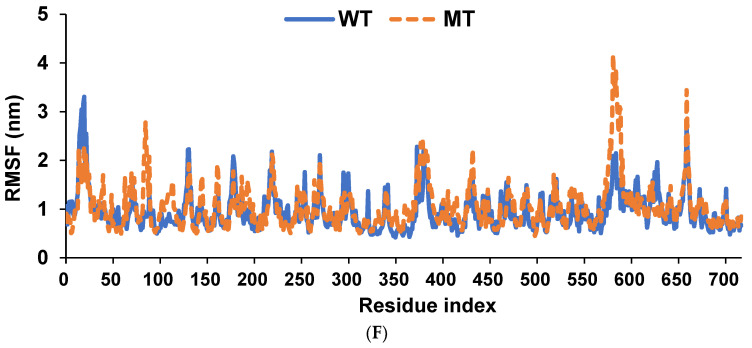
Molecular docking and molecular dynamic simulation (MDS) of rugosin A on growth differentiation factor-5 (*GDF-5*) rs224331 (Ala276Ser) wild and mutated types. (**A**) Molecular docking of rugosin A on *GDF5* rs224331 wild type. (**B**) The interaction force between rugosin A on *GDF5* rs224331 wild type. (**C**) Molecular docking of rugosin A on *GDF5* rs224331 mutated type. (**D**) The interaction force between rugosin A on *GDF5* rs224331 mutated type. (**E**) RMSD of rugosin A on *GDF5* rs224331 wild and mutated types. (**F**) RMSF of rugosin A on *GDF5* rs224331 wild and mutated types.

The wild and mutated *GDF5*_rs224331 exhibited somewhat different binding free energy to food agents (Table 4). Among them, rugosin A is shown in Figure 4 as an example. The wild type of *GDF5*_rs224331 had a decreased binding free energy (<−10.7 kcal/mol) to hydrolyzable tannins such as stachyurin, lambertianin B, sanguiin H6, lambertianin A, mongolicain A, casuariin, punicacortein D, rugosin A, rugosin E, valolaginic acid, rugosin D, cinnamtannin II, eugenigrandin A, rugosin A, Chinese tannin, and gemin D (Table 4). However, some hydrolyzable tannins, including rugosin A, rugosin D, rugosin E, and valolaginic acid, increased binding free energy to the mutated type, indicating that they had a lower binding affinity to mutated *GDF5*. The binding energy between the wild-type GDF5 protein and rugosin A with hydrogen bond in Figure 4A and the pink and green parts indicated hydrogen donor and acceptor, respectively. Their binding position and intermolecular force are shown in the two-dimensional picture in Figure 4B. The binding and interactions of binding affinity between rugosin A and the mutated type of *GDF5* are also shown in Figure 4C,D. 

Figure 4E,F showed the root mean square deviation (RMSD) and root mean square fluctuation (RMSF) for *GDF5* wild and mutated types binding to rugosin A. RMSD for *GDF5* wild type binding with rugosin A was sustained close to 3 Å during 100 nanoseconds. RMSF for *GDF5* wild-type binding with rugosin A also did not exceed 3 nm, except at the 580 residue index in the RMSF graph. These results suggest that rugosin A stably bound to the *GDF5* wild type. 

### 3.8. Association of PRS with the Risk of Metabolic Syndrome and Its Components

Height was greater with PRS in the ascending order. The PRS for the four-SNP model was not related to body composition, including BMI, waist circumferences (Table 5), SMI, and fat mass, except height. Osteoporosis and arthritis showed no significant change with changes in PRS. However, PRS was inversely related to WBC, an immunity index (Table 5). The PRS was inversely linked to MetS, blood HbA1c, and serum glucose concentrations, but was not significantly associated with insulin resistance. However, lipid profiles, blood pressure, and serum ALT and AST concentrations were not associated with PRS (Table 5). Therefore, the association of height-related PRS with glucose metabolism is likely related to insulin secretion. However, serum HDL, LDL, and triglyceride concentrations did not differ among the PRS groups. SBP and DBP showed a similar trend (Table 5). However, serum ALT concentrations but not AST were lower in the High-PRS compared to the Low-PRS groups and were inversely associated with PRS (Table 5). 

### 3.9. Interaction between PRS and Lifestyle Factors for Adult Height

Among the lifestyle factors, energy intake and rice-main diet interacted with PRS to influence height (*p* = 0.0078 and *p* = 0.0095) (Table 6). In people with high energy intake, PRS was positively related to height by 1.414 times but not in those with low energy intake. PRS interacted with the rice-main diet to affect height among the dietary patterns (Table 6). In adults with a low rice-main diet, PRS was positively linked to height by 1.318 times but not in those with a high rice-main diet. The results suggested that the genetic impact for adult height was not shown to be related to low energy intake, mainly containing rice. 

## 4. Discussion

The adult height peaked in the 20s and gradually reduced after 30 years of age. Although the loss of height varies from individual to individual, various factors are involved in the decrement. The impact of genetics explains about 10% of the height variation among people of East Asian, Hispanic, African, and South Asian ancestry [30]. An average adult loses about two centimeters in height every decade after age 30. Women tend to lose more height than men due to an accelerated rate of bone loss during menopause [31]. The loss of height can also be accelerated by certain health conditions such as osteoporosis, poor nutrition, lack of exercise, and some medical therapies such as chemotherapy and radiation therapy [32]. Therefore, the present study explored the genetic variants for tall stature using adult height and adjusting for covariates related to metabolic syndrome. Our results indicated that the selected genetic variants could explain the genetic impact on tall stature in Asians. 

Short height in adults is linked to an increased risk of MetS. Previous studies have demonstrated that being short is associated with higher body fat, higher triglycerides, lower serum HDL cholesterol concentrations, and an increased risk of hypertension and type 2 diabetes, all of which are components of MetS [33,34]. However, tall stature is associated with obesity during childhood [35]. The association between stature and MetS and the impact of genetics remains unclear. The present study exhibited that tall stature was inversely linked to MetS, its components, CVD, fat and skeletal muscle mass, serum ALT and AST concentrations, and arthritis, but not osteoporosis, after adjusting height-related parameters. It was related to increased insulin resistance, which might be a primary factor for the inverse relationship between tall stature and the risk of MetS and CVD. An earlier German study demonstrated that an additional height of 10 cm is associated with a 41% and 33% lower risk of type 2 diabetes among men and women, respectively. Moreover, the association of height with a lower risk of type 2 diabetes was also seen in overweight or obese men and women (36% in men and 30% in women), although the decrease in risk was lower than that seen in normal-weight adults [33]. Therefore, increased insulin resistance in short stature elevated MetS risk, which was consistent with the results of the current study. 

Common genetic variants significantly influence height. Genetic variants affect the trait by the cumulative effects of many alleles at multiple loci rather than a single genetic variant. In European ancestries, one of the most commonly identified variants is the rs1042725 variant in the *HMGA2* gene, which is associated with a 0.4 cm increase in height [36,37]. However, the present study did not include *HMGA2*_rs1042725 as a height-related genetic variant in Asians, indicating that Asians have different genetic variants for height traits. In the genetic investigation of the anthropometric traits (GIANT) consortium, 12,111 common SNPs are associated with the height trait, accounting for 10–40% of all height variations depending on the person’s ancestry [30]. Previous studies have shown that genetic variants for the height trait are somewhat different between Asians and Caucasians. The genetic variants in the loci of *LCORL*, *CABLES1*, *CDK10*, *ZBTB38*, *ZNF638*, and *TSEN15* are linked to stature in Han Chinese from the Beijing study [9]. The polygenic loci of LCORL, *DIS3L2*, *EFEMP1*, *ZBTB38*, high mobility group AT-hook 1 (*HMGA1*), citrate synthase (*CS)*, and *GDF5* are also shown to be linked to stature in Taiwanese [38] and Japanese [39]. Some genetic variants related to height in Asians (*ADAMTSL3*, *ZBTB38*, *LCORL*, *DIS3L2*, and *GDF5*) overlapped with those in the present study. Therefore, polygenic variants linked to height vary according to ethnicity. 

Height is inversely associated with insulin resistance, and the waist-to-height ratio is used to predict insulin resistance [40]. The present study also demonstrated that height and waist-to-height ratio were inversely associated with insulin resistance. Some polygenic variants related to height, such as *IGF1R*, *GDF5*, *DIS3L2*, and *ADAMTSL3*, have been reported to modulate serum glucose and insulin concentrations, potentially [41,42]. Interestingly, the PRS of the common polygenic variants related to height was associated with MetS and its components, fasting serum glucose and blood HbA1c concentrations, but not insulin resistance in the present study. The PRS was also associated with MetS, a consequence of insulin resistance. However, PRS was not associated with insulin resistance. PRS could be associated with insufficient insulin secretion, commonly seen in Asians with type 2 diabetes, and it suggests that height-related genetic variants could be associated with insulin secretion. 

Genetic variants affect gene function by modifying gene expression and/or resulting in catalytic activity by altering protein conformation, especially in missense mutations. Gene expression having risk alleles is different in different tissue types. In the present study, the polygenic variants selected for stature were expressed mainly in the tibial nerves. In the tibial nerve, the risk alleles of *NCAPG*, *LCORL*, *IGF1*, and *GDF5* had a lower expression than the non-risk allele, while the risk allele of *ADAMTSL3* showed a higher expression than the non-risk allele. *NCAPG* is responsible for condensing and stabilizing chromosomes during mitosis and meiosis. It is also involved in the carcinogenesis and progression of tumors [43]. *LCORL* is a transcription factor involved in spermatogenesis related to skeletal frame size and adult height [44,45]. The *NCAPG-LCORL* locus is associated with body growth and feed intake in cattle [46]. The IGF1R → insulin/phosphatidylinositol-3 kinase (PI3K) → protein kinase B (Akt) signaling pathway is a critical pathway for the growth of long bones [47]. The mutation in c.926C > T of *IGF1R* is involved in severe short stature in Chinese [48]. However, it did not show a significant relationship to adult stature in the present study. On the other hand, the rs2871865 variant in the intron of the *IGF1R* was significantly associated with stature. Its expression of the risk allele was lower than that of the non-risk allele in the tibial nerve. These results suggest that the genetic variations for height were mainly expressed in the tibial nerve, which receives the message from the brain for movement of the legs, feet, and toes. The tibial nerve conduction velocity is reported to be inversely associated with height [49]. 

Among the selected genetic variants for height, the expression of *GDF5* was the most prominent and has been revealed to be involved in height and osteoarthritis. *GDF5* variants are expressed in tissues such as the tibial nerve, brain, pituitary, thyroid, and adipose tissue. Its risk allele lowered the GDF5 protein expression more than the non-risk allele [50,51,52]. In addition, *GDF5*_rs224331 could affect the catalytic activity since the SNP site is a missense mutation. Hydrolyzable tannins are reported to increase the expression of collagen I, a primary component of the extracellular matrix found in the bones, to improve bone growth and ameliorate osteoarthritis [53]. They also stimulate osteoblast proliferation and differentiation and suppress osteoclast activity [54,55]. Hydrolyzable tannins have been demonstrated to have anti-inflammatory and antioxidant properties, which may help in bone growth [56]. It suggests hydrolyzable tannins may affect GDF5 to modulate bone growth and osteoarthritis. The present study demonstrated that some bioactive compounds had a decreased binding free energy with the *GDF5*_rs224331 wild type. Hydrolyzable tannins such as stachyurin, lambertianin A and B, sanguiin H6, mongolicain A, casuariin, punicacortein D, cinnamtannin II, eugenigrandin A, Chinese tannin, and gemin D had a lower binding free energy. However, the mutated *GDF5* had a decreased binding affinity (increased binding free energy) with rugosin E, rugosin A, rugosin D, and valolaginic acid. It indicated that *GDF5*_rs224331 might achieve its activity by modifying binding free energy. Bioactive compound effects can be altered with the *GDF5*_rs224331 mutation. Further experimental studies are needed to confirm this. 

The study is novel in showing that polygenic variants are involved in height through SNP–SNP interactions in Asians and were also associated with immunity and glucose metabolism. Interestingly, their expressions were mainly linked to the tibial nerve. *GDF5*_rs224331 is a missense mutation; its binding affinity to some hydrolyzable tannins, such as rugosin E, rugosin A, rugosin D, and valolaginic acid, was lower in the wild type than the mutated one. Although further studies are needed, it can be suggested that bone growth is related to modulating the expression and binding affinity of *GDF5*_rs224331. However, the limitations of the present study were as follows: First, adult height was used for estimating genetic impact, which could provide good results since it was adjusted for the covariates affecting reduction in height after age 30. Second, it was conducted as a cross-sectional study; although the sample size was large (n = 58,701), the specialists gathered the samples uniformly from the volunteers. Third, data on usual food intake were gathered using the SQFFQ, in which usual food intake could be underestimated or overestimated, although it was designed for Koreans and validated with 3-day food records for four seasons. 

## 5. Conclusions

The genetic impact on tall stature was found to be 1.29 times with the four-SNP model, including *ZBTB38*_rs6762722, *PAIP2B*_rs13034890, *CPZ*_rs3756173, and *LTBP1*_rs4630744. Although the *GDF*_rs224331 and *IGF-1R*_rs2871865 were significantly associated with tall stature, they did not interact with other SNPs to lower the adjusted OR in model 7. Furthermore, the PRS was inversely associated with MetS, hyperglycemia, and WBC risk. The SNPs in the model were explicitly expressed in the tibial nerve, which is associated with increased height. Hydrolyzable tannins lowered the binding free energy with the wild type of *GDF5*_rs143384. However, some hydrolyzable tannins (rugosin E, rugosin A, rugosin D, and valolaginic acid) did not decrease binding free energy with the mutated gene. Therefore, the genetic variants for tall stature may modulate not only height growth but also MetS, glucose metabolism, and immunity by altering the gene expression and/or their activity. Adults with PRS for short-stature need to be more cautious of the MetS risk. These results can be used in precision nutrition after further clinical study has been conducted. 

## Figures and Tables

**Table 1:** Demographic characteristics of the participants according to gender and adult height.

	Men		Women	
	Short-Stature (n = 17,305)	Tall-Stature (n = 2988)	Short-Stature (n = 33,860)	Tall-Stature (n = 4548)
Age (years)	56.4 ± 0.06 ^b^	53.2 ± 0.18 ^a^	52.8 ± 0.04 ^c^	50.2 ± 0.11 ^c^***^+++^
Gender (%)	33.82	39.65 ^‡‡‡^	66.18	60.35 ^‡‡‡^
Education ≤Middle school High school ≥College	1654 (15.1) 8261 (75.4) 1045 (9.53)	99 (6.52) ^‡‡‡^ 1173 (77.3) 246 (16.2)	6409 (23.5) 19,373 (71.1) 1486 (5.45)	329 (10.5) ^‡‡‡^ 2498 (80.0) 296 (9.48)
Income ≤$2000 $2000–4000 >$4000	1483 (9.03) 7184 (43.7) 7764 (47.3)	268 (10.7) ^‡‡‡^ 1125 (44.9) 1111 (44.4)	3911 (12.3) 14,278 (44.9) 13,599 (42.8)	261 (6.02) ^‡‡‡^ 1707 (39.3) 2371(54.6)
Former smoking (%) Smoking (%)	2237 (12.9) 1447 (8.36)	349 (11.7) 275 (9.21)	100 (0.30) 172 (0.51)	17 (0.37) 19 (0.42)
Physical exercise (%)	10,168 (59.0)	1784 (59.9)	17,579 (52.1)	2445 (53.8) ^‡^
Alcohol (g)	35.2 ± 0.38 ^b^	39.8 ± 0.88 ^a^	5.24 ± 0.27 ^b^	5.89 ± 0.71 ^b^***^+++##^
Energy intake (EER %)	89.4 ± 0.26 ^d^	93.3 ± 0.58 ^c^	99.1 ± 0.18 ^b^	101.6 ± 0.47 ^a^***^###^
Carbohydrates (En%)	71.3 ± 0.06 ^b^	71.2 ± 0.13 ^b^	72.0 ± 0.04 ^a^	71.6 ± 0.11 ^b^***^+^
Fat (En%)	14.2 ± 0.04 ^a^	14.3 ± 0.10 ^a^	13.7 ± 0.03 ^b^	14.1 ± 0.08 ^a^***^++#^
Protein (En%)	13.3 ± 0.02 ^b^	13.3 ± 0.05 ^b^	13.5 ± 0.02 ^a^	13.5 ± 0.04 ^a^***
Fiber (g)	15.2 ± 0.08 ^b^	15.8 ± 0.18 ^a^	14.3 ± 0.06 ^c^	14.6 ± 0.15 ^c^***^+++^
Calcium (mg)	411 ± 2.15 ^d^	427 ± 4.89 ^c^	456 ± 1.50 ^b^	471 ± 3.95 ^a^***^+++^
Vitamin C (mg)	95.1 ± 0.56 ^d^	98.9 ± 1.28 ^c^	110 ± 0.39 ^b^	113 ± 1.04 ^a^***^++^
Vitamin D (ug)	5.59 ± 0.05 ^d^	5.89 ± 0.11 ^c^	6.81 ± 0.03 ^b^	7.09 ± 0.09 ^a^***^+++^
DII (scores)	−13.8 ± 0.39	−14.2 ± 0.89	−13.4 ± 0.27	−15.5 ± 0.72
Flavonoids (mg)	32.1 ± 0.27 ^b^	32.8 ± 0.61 ^b^	41.5 ± 0.19 ^a^	42.6 ± 0.49 ^a^***^+^
KBD (%)	6832 (39.5)	1269 (42.5) ^‡‡^	10,059 (29.7)	1409 (31.0)
PBD (%)	3551 (20.5)	647 (21.7)	13,316 (39.3)	2062 (45.3) ^‡‡‡^
WSD (%)	8619 (49.8)	1812 (60.6) ^‡‡‡^	11,155 (32.9)	1966 (43.2) ^‡‡‡^
RMD (%)	5482 (31.7)	983 (32.9)	11,480 (33.9)	1626 (35.8) ^‡^
Coffee intake (g/day)	4.23 ± 0.03 ^a^	4.25 ± 0.06 ^a^	3.34 ± 0.02 ^c^	3.48 ± 0.05 ^b^***^+^
Tea (g/day)	43.5 ± 0.71 ^ab^	47.7 ± 1.63 ^a^	43.2 ± 0.50 ^b^	42.2 ± 1.32 ^b^***

Values represent adjusted means and standard errors for continuous variables and the number and percentage for categorical variables. Values represent adjusted odd ratios and 95% confidence intervals. Covariates included age, sex, weight at age 18, education, income, energy intake (percentage of estimated energy requirement), residence areas, daily activity, alcohol intake, and smoking status. EER, estimated energy requirement; En%, energy percentage; DII, dietary inflammation index; KBD, Korean-balanced diet; PBD, plant-based diet; WSD, Western-style diet; RMD, rice-main diet. *** Significant differences by gender at *p* < 0.001. ^+^ Significant differences by height at *p* < 0.05, ^++^ at *p* < 0.01, ^+++^ *p* < 0.001. ^#^ Significant interaction between gender and height at *p* < 0.05, ^##^ at *p* < 0.01, ^###^ *p* < 0.001. ^‡^ Significantly different from the control group in χ^2^ test in each gender at *p* < 0.05, ^‡‡^ at *p* < 0.01, ^‡‡‡^ at *p* < 0.001. ^a, b, c, d^ Different superscript letters indicated significant differences among the groups in Tukey’s test at *p* < 0.05.

**Table 2:** Adjusted means and odds ratio (ORs) of metabolic syndrome-related parameters according to gender and adult height.

	Men		Women		
	Short- Stature (n = 17,305)	Tall- Stature (n = 2988)	Short- Stature (n = 33,860)	Tall-Stature (n = 4548)	Adjusted OR and 95% CI
Height (cm) ^1^	167.3 ± 0.03 ^b^	177.2 ± 0.08 ^a^	155.5 ± 0.02 ^d^	164.7 ± 0.06 ^c^***^+++###^	
BMI (kg/m^2^) ^2^	24.5 ± 0.04 ^a^	24.6 ± 0.07 ^a^	23.6 ± 0.03 ^b^	23.0 ± 0.06 ^c^***^+++###^	0.908 (0.857–0.962)
Waist (cm) ^3^	80.1 ± 0.07 ^c^	76.9 ± 0.13 ^a^	81.5 ± 0.05 ^b^	78.7 ± 0.10 ^d^***^+++#^	0.327 (0.299–0.357)
Weight at age 18 (kg) ^4^	55.4 ± 0.14	56.1 ± 0.31	55.0 ± 0.11	55.1 ± 0.20 ***	1.025 (0.956–1.099)
SMI (kg/m) ^5^	7.34 ± 0.004 ^a^	7.04 ± 0.010 ^b^	6.90 ± 0.003 ^c^	6.46 ± 0.006 ^d^***^+++#^	0.402 (0.376–0.430)
Fat mass (%) ^6^	20.4 ± 0.02 ^c^	17.3 ± 0.05 ^d^	32.8 ± 0.02 ^a^	30.1 ± 0.04 ^b^***^+++###^	0.479 (0.445–0.515)
WBC (10^9^/L) ^7^	5.79 ± 0.02 ^a^	5.61 ± 0.04 ^b^	5.67 ± 0.01 ^b^	5.60 ± 0.03 ^c^*^+++#^	0.813 (0.767–0.861)
hs-CRP (mg/dL) ^8^	0.14 ± 0.004 ^ab^	0.16 ± 0.009 ^a^	0.14 ± 0.003 ^ab^	0.12 ± 0.007 ^b^***	0.784 (0.612–1.005)
MetS ^9^	3005 (17.4)	593 (19.9) ^‡‡^	4275 (12.6)	427 (9.39) ^‡‡‡^	0.494 (0.452–0.540)
CVD ^9^	1107 (6.41) ^‡‡^	109 (3.65)	1030 (3.05)	68 (1.50) ^‡‡‡^	0.669 (0.563–0.794)
Glucose (mg/dL) ^10^	96.38 ± 0.27 ^a^	93.4 ± 0.55 ^b^	95.4 ± 0.19 ^a^	93.4 ± 0.42 ^b+++^	0.718 (0.657–0.785)
HbA1c (%) ^11^	5.63 ± 0.01 ^b^	5.49 ± 0.03 ^c^	5.79 ± 0.01 ^a^	5.68 ± 0.02 ^b^***^+++^	0.659 (0.569–0.763)
Insulin resistance (%) ^9^	1955 (11.3)	347 (12.0)	20,66 (6.1)	226 (4.97) ^‡‡^	0.542 (0.487–0.603)
Total cholesterol (mg/dL) ^12^	189 ± 0.35 ^c^	190 ± 0.71 ^c^	202 ± 0.23 ^a^	198 ± 0.56 ^b^***^+++###^	0.707 (0.658–0.758)
HDL (mg/dL) ^13^	52.2 ± 0.18 ^b^	53.9 ± 0.36 ^c^	55.1 ± 0.13 ^a^	57.3 ± 0.24 ^a^***^++###^	1.330 (1.249–1.415)
LDL (mg/dL) ^14^	112 ± 0.45 ^c^	112 ± 0.94 ^c^	122 ± 0.33 ^a^	117 ± 0.71 ^b^***^+++###^	0.702 (0.646–0.763)
TG (mg/dL) ^15^	120 ± 1.11 ^b^	105 ± 2.29 ^c^	127 ± 0.81 ^a^	109 ± 1.74 ^c^**^+++^	0.633 (0.594–0.675)
SBP (mmHg) ^16^	123 ± 0.20 ^a^	120 ± 0.40 ^b^	123 ± 0.14 ^a^	120 ± 0.31 ^b+++^	0.749 (0.704–0.796)
DBP (mmHg) ^17^	76.9 ± 0.13 ^a^	75.1 ± 0.27 ^b^	75.5 ± 0.09 ^b^	73.9 ± 0.20 ^c^***^++#^	0.698 (0.633–0.770)
AST (IU/L) ^18^	25.1 ± 0.20 ^a^	25.1 ± 0.45 ^a^	23.1 ± 0.14 ^b^	22.5 ± 0.36 ^b^***	0.633 (0.550–0.729)
ALT (IU/L) ^19^	24.5 ± 0.16 ^a^	23.6 ± 0.34 ^b^	23.5 ± 0.12 ^b^	22.4 ± 0.25 ^c^**^+++^	0.531 (0.485–0.582)
Egfr ^20^	84.4 ± 0.21 ^b^	84.1 ± 0.43 ^b^	86.6 ± 0.15 ^a^	85.3 ± 0.33 ^b^***^++^	0.940 (0.867–1.020)
Arthritis (N, %) ^9^	698 (4.04)	111 (3.72)	3995 (11.8)	326 (7.17) ^‡‡‡^	0.868 (0.774–0.973)
Osteoporosis (N, Yes%) ^9^	117 (0.68)	16 (0.54)	2779 (8.22)	162(3.56) ^‡‡‡^	0.882 (0.740–1.051)

CI, confidence interval; SBP, systolic blood pressure; DBP, diastolic blood pressure; AST, aspartate aminotransferase; ALT, alanine aminotransferase; TG, triglyceride. The high-height group was ≥175 for men and ≥163 for women. Adjusting for covariates including weight, gender, age, weight at age 18, education, income, total activity, energy intake, alcohol intake, residence area, and smoking status. The cutoff points of the reference for logistic regression were as following: ^1^ <172.5 cm for men and <160 cm for women; ^2^ <25 kg/m^2^ for BMI; ^3^ <90 cm for men and 85 cm for women waist circumferences; ^4^ <60 kg for men and <59 kg for women; ^5^ <7.6 kg/m in men and 5.4 kg/m in women for SMI in skeletal muscle index (SMI defined as appendicular skeletal muscle mass/height); ^6^ <25% for men and 30% for women for fat mass; ^7^ <4.0 × 10^9^ counts/L WBC; ^8^ <0.5 mg/dL serum high-sensitive C-reactive protein (hs-CRP) concentrations; ^9^ disease incidence; ^10^ <126 mL/dL fasting serum glucose plus diabetic drug intake; ^11^ <6.5% blood HbA1c plus diabetic drug intake; ^12^ <230 mg/dL serum total cholesterol concentrations; ^13^ >40 mg/dL for men and 50 mg/dL for women serum HDL cholesterol; ^14^ <160 mg/dL serum total cholesterol concentrations; ^15^ <150 mg/dL serum triglyceride concentrations; ^16^ <140 mmHg SBP, ^17^ <90 mmHg DBP plus hypertension medication; ^18^ <40 U/L aspartate aminotransferase; ^19^ <35 U/L alanine aminotransferase; ^20^ <70 mL/min/1.73 m^2^ estimated glomerular filtration rate (eGFR). * Significant differences by gender at *p* < 0.05, ** at *p* < 0.01, *** at *p* < 0.001. ^++^ Significant differences by height at *p* < 0.01, ^+++^ *p* < 0.001. ^#^ Significant interaction between gender and height at *p* < 0.05, ^###^ at *p* < 0.001. ^‡‡^ Significantly different from the control group in χ^2^ test in each gender at *p* < 0.01, ^‡‡‡^ at *p* < 0.001. ^a, b, c, d^ Different superscript letters indicated significant differences among the groups in Tukey’s test at *p* < 0.05.

**Table 3:** Characteristics of genetic variants related to adult height from GMDR analysis.

CHR ^1^	SNP ^2^	Base Pair	A1 ^3^	A2 ^4^	OR ^5^	SE ^6^	*p* for City ^7^	*p* for Asan + Nong ^8^	MAF ^9^	*p* for HWE ^10^	Gene Names	Location
2	rs4630744	33461375	G	A	1.105	0.01906	1.53 × 10^−7^	0.02458	0.3792	0.4099	*LTBP1*	Intron
2	rs13034890	71430542	T	C	0.9086	0.01886	3.72 × 10^−7^	0.0014	0.4567	0.6295	*PAIP2B*	Intron
2	rs1249260	233046182	C	T	1.172	0.01877	2.99 × 10^−17^	0.00016	0.4515	0.7705	*DIS3L2*	Downstream
3	rs6762722	141145216	G	A	1.177	0.02095	6.65 × 10^−15^	0.000749	0.2546	0.5644	*ZBTB38*	Intron
4	rs3756173	8598698	T	C	0.9058	0.01917	2.48 × 10^−7^	0.000515	0.4135	0.5123	*CPZ*	Intron
4	rs2074974	17812615	C	A	0.8989	0.01884	1.57 × 10^−8^	0.00103	0.461	0.8746	*NCAPG*	5′ UTR
4	rs7700107	17880416	C	A	0.8112	0.02421	5.34 × 10^−18^	6.78 × 10^−5^	0.2072	0.1078	*LCORL*	Downstream
15	rs1600640	84603034	T	G	0.8711	0.02363	5.18 × 10^−9^	0.000252	0.2115	0.5692	*ADAMTSL3*	Intron
15	rs2871865	99194896	G	C	0.8078	0.03676	6.34 × 10^−9^	0.00673	0.0800	0.0640	*IGF1R*	Intron
20	rs224331	34022387	A	C	1.191	0.02073	3.13 × 10^−16^	0.000956	0.2683	0.8747	*GDF5*	Missense (Ala276Ser)

^1^ Chromosome; ^2^ Single nucleotide polymorphism; ^3^ Minor allele; ^4^ Major allele^; 5^ Odds ratio (OR) for city cohort; ^6^ Standard error; ^7^ *p*-value for OR after adjusting for age, gender, residence area, survey year, body mass index, daily energy intake, education and income in the hospital-based cohort (case: n = 17,545; control: n = 36,283); ^8^ *p*-value for OR after adjusting for covariates in the Ansan/Ansung cohort (case: n = 1657; control: n = 3245); ^9^ Minor allele frequency; ^10^ Hardy–Weinberg equilibrium. *LTBP1*, latent transforming growth factor beta binding protein-1; *PAIP2B*, poly(A)-binding protein interacting protein-2B; *DIS3L2*, DIS3 like 3′-5′ exoribonuclease-2; *ZBTB38*, zinc finger and BTB domain containing 38; *CPZ*, carboxypeptidase Z; *NCAPG*, non-SMC condensin I complex subunit G; *LCORL*, ligand-dependent nuclear receptor corepressor like; *ADAMTSL3*, a disintegrin and metalloproteinase with thrombospondin motifs-3; *IGF1R*, insulin-like growth factor-1 receptor; *GDF5*, growth differentiation factor-5.

**Table 4:** Biding energy of hydrolyzable tannins to GDF5 wild type and mutated one in rs224331 (unit: kcal/mol).

Compounds	Wide Type	Mutated Type
Stachyurin	−13.7	−13.8
Lambertianin B	−13.3	−13.3
Sanguiin H6	−13.2	−13.3
Lambertianin A	−13.2	−13.3
Mongolicain A	−12.9	−12.3
Casuariin	−12.7	−12.7
Punicacortein D	−12.5	−11.9
Rugosin E	−12.2	−5
Valolaginic acid	−12.1	−9.7
Rugosin D	−12	−5
Cinnamtannin II	−11.8	−11.8
Eugenigrandin A	−11.7	−11.8
Rugosin A	−11	−5.1
Chinese tannin	−10.9	−11
Gemin D	−10.7	−10.6

**Table 5:** Adjusted means and odds ratio (ORs) of metabolic syndrome (metS)-related parameters according to polygenic risk score (PRS) of the four-SNP model generated from adult height.

	Low-PRS (n = 6107)	Middle-PRS (n = 29,668)	High-PRS (n = 22,926)	Adjusted ORs and 95 CI
Height (cm) ^1^	160.4 ± 0.07 ^c^	160.7 ± 0.03 ^b^	160.9 ± 0.04 ^a^***	1.293 (1.127–1.381)
BMI (kg/m^2^) ^2^	23.8 ± 0.04	23.8 ± 0.02	23.9 ± 0.02	1.054 (0.987–1.124)
Waist (cm) ^3^	80.6 ± 0.10	80.7 ± 0.05	80.7 ± 0.05	0.975 (0.883–1.077)
Weight at 18 ^4^	54.8 ± 0.11 ^b^	55.2 ± 0.05 ^a^	55.1 ± 0.06 ^ab^	1.059 (0.994–1.129)
SMI ^5^	7.05 ± 0.008 ^a^	7.01 ± 0.004 ^b^	6.98 ± 0.004 ^c^***	0.960 (0.896–1.029)
Fat mass ^6^	28.3 ± 0.04	28.3 ± 0.02	28.4 ± 0.02	0.959 (0.896–1.027)
WBC (10^9^/L) ^7^	5.78 ± 0.02 ^a^	5.70 ± 0.01 ^b^	5.68 ± 0.01 ^b^**	0.894 (0.837–0.954)
Serum hs-CRP (mg/dL) ^8^	0.152 ± 0.006 ^a^	0.136 ± 0.003 ^b^	0.141 ± 0.003 ^ab^*	0.862 (0.675–1.100)
MetS ^9^	883 (14.5)	4162 (14.0)	3255 (14.2)	0.894 (0.815–0.982)
Serum glucose (mg/dL) ^10^	95.5 ± 0.26	95.1 ± 0.12	95.0 ± 0.14	0.905 (0.828–0.990)
Blood HbA1c (%) ^11^	5.73 ± 0.01 ^a^	5.71 ± 0.01 ^b^	5.71 ± 0.01 ^b^*	0.851 (0.740–0.980)
Insulin resistance (%)	1955 (11.3)	347 (12.0)	2066 (6.1)	0.953 (0.854–1.064)
Serum total cholesterol (mg/dL) ^12^	197 ± 0.48	197 ± 0.22	197 ± 0.25	0.940 (0.875–1.010)
Serum HDL (mg/dL) ^13^	53.6 ± 0.17	53.8 ± 0.08	53.8 ± 0.09	1.047 (0.978–1.120)
Serum LDL (mg/dL) ^14^	119 ± 0.44	119 ± 0.20	119 ± 0.23	0.960 (0.883–1.043)
Serum TG (mg/dL) ^15^	126 ± 1.11	125 ± 0.50	124 ± 0.57	0.981 (0.917–1.048)
SBP (mmHg) _16_	122 ± 0.19	122 ± 0.08	123 ± 0.10	1.050 (0.984–1.121)
DBP (mmHg) ^17^	75.6 ± 0.12	75.6 ± 0.06	75.8 ± 0.06	1.020 (0.919–1.132)
Serum AST (IU/L) ^18^	24.7 ± 0.31 ^a^	23.7 ± 0.14 ^b^	23.5 ± 0.16 ^b^	0.875 (0.755–1.014)
Serum ALT (IU/L) ^19^	23.4 ± 0.30 ^a^	22.4 ± 0.14 ^b^	22.1 ± 0.16 ^b^	0.893 (0.812–0.981)
Egfr ^20^	86.5 ± 0.20	86.0 ± 0.09	86.1 ± 0.11	1.059 (0.893–1.257)
Arthritis (N, %)	549 (9.0)	2619 (8.84)	1962 (8.57)	0.920 (0.825–1.026)
Osteoporosis (N, Yes%)	344 (5.64)	1568 (5.29)	1162 (5.08)	0.920 (0.800–1.057)

CI, confidence interval; SBP, systolic blood pressure; DBP, diastolic blood pressure; AST, aspartate aminotransferase; ALT, alanine aminotransferase; TG, triglyceride; N, the number of participants. The high-height group was ≥175 for men and ≥163 for women. Adjusting for covariates including age, gender, weight, body weight at age 18, education, income, total activity, energy intake, alcohol intake, residence area, and smoking status. PRS with four SNPs of the best GMDR model was divided into three categories according to the number of the risk alleles: ≤3, 4–5, and ≥6 into Low-PRS, Middle-PRS, and High-PRS, respectively. The cutoff points of the reference for logistic regression were as following: ^1^ <172.5 cm for men and <160 cm for women; ^2^ <25 kg/m^2^ for BMI; ^3^ <90 cm for men and 85 cm for women waist circumferences; ^4^ <60 kg for men and <59 kg for women; ^5^ <7.6 kg/m in men and 5.4 kg/m in women for SMI in skeletal muscle index (SMI defined as appendicular skeletal muscle mass/height); ^6^ <25% for men and 30% for women for fat mass; ^7^ <4.0 × 10^9^ counts/L WBC; ^8^ <0.5 mg/dL serum high-sensitive C-reactive protein (hs-CRP) concentrations; ^9^ metabolic syndrome; ^10^ <126 mL/dL fasting serum glucose plus diabetic drug intake; ^11^ <6.5% HbA1c plus diabetic drug intake; ^12^ <230 mg/dL plasma total cholesterol concentrations; ^13^ >40 mg/dL for men and 50 mg/dL for women plasma HDL cholesterol; ^14^ <160 mg/dL plasma total cholesterol concentrations; ^15^ <150 mg/dL plasma triglyceride concentrations; ^16^ <140 mmHg SBP, ^17^ <90 mmHg DBP plus hypertension medication; ^18^ <40 U/L aspartate aminotransferase; ^19^ <35 U/L alanine aminotransferase; ^20^ <70 mL/min/1.73 m^2^ estimated glomerular filtration rate (eGFR). * Significant differences by PRS at *p* < 0.05, ** at *p* < 0.01, *** *p* < 0.001. ^a, b, c^ Different superscript letters indicated significant differences among the groups in Tukey’s test at *p* < 0.05.

**Table 6:** Adjusted odds ratios for adult height risk by polygenetic risk scores (PRS) of the 4-SNP model for SNP-SNP interaction after covariate adjustments according to the patterns of lifestyles.

	Low-PRS (n = 14,420)	Middle-PRS (n = 21,641)	High-PRS (n = 4201)	Gene-Nutrient Interaction *p* Value
Low energy ^1^ High energy	1	1.032 (0.914–1.165) 1.253 (1.075–1.461)	1.130 (0.999–1.278) 1.414 (1.210–1.652)	0.0078
Low KBD ^2^ High KBD	1	1.092 (0.971–1.228) 1.158 (0.984–1.362)	1.183 (1.050–1.333) 1.334 (1.132–1.574)	0.0923
Low PBD ^2^ High PBD	1	1.056 (0.938–1.188) 1.220 (1.039–1.432)	1.157 (1.026–1.305) 1.377 (1.170–1.620)	0.0567
Low WSD ^2^ High WSD	1	1.070 (0.937–1.222) 1.163 (1.015–1.332)	1.188 (1.038–1.359) 1.282 (1.117–1.472)	0.1898
Low RMD ^2^ High RMD	1	1.157 (1.027–1.303) 1.041 (0.888–1.220)	1.318 (1.168–1.486) 1.092 (0.929–1.284)	0.0095
Low alcohol ^3^ High alcohol	1	1.143 (1.003–1.303) 1.081 (0.941–1.241)	1.286 (1.125–1.469) 1.175 (1.021–1.353)	0.6960
Low exercise ^4^ High exercise	1	1.037 (0.901–1.194) 1.173 (1.031–1.335)	1.202 (1.042–1.386) 1.253 (1.099–1.429)	0.2233
Non-smoking Former smoking +smoking	1	1.124 (1.003–1.260) 1.095 (0.920–1.302)	1.225 (1.091–1.375) 1.260 (1.057–1.502)	0.0547

Values represent adjusted odd ratios and 95% confidence intervals. Covariates included age, sex, education, income, energy intake (percentage of estimated energy requirement), residence areas, daily activity, alcohol intake, and smoking status. PRS with four SNPs of the best GMDR model was divided into three categories according to the number of the risk alleles: ≤3, 4–5, and ≥6 into Low-PRS, Middle-PRS, and High-PRS, respectively. ^1^ <Estimated energy requirement defined in dietary reference index; ^2^ <75th percentiles; ^3^ <20g daily alcohol intake; ^4^ <moderate exercise for 150 min/day.

## Data Availability

The raw data involved in this study will be available by the corresponding author to any qualified researcher.

## Supplementary Materials

The following supporting information can be downloaded at: https://www.mdpi.com/article/10.3390/nu15071764/s1. Table S1: Generalized multifactor dimensionality reduction (GMDR) results of multi-locus interaction with genes related to adult height; Figure S1: Distribution of genetic variants for tall stature by a genome-wide association study.
