# Supplementary material for: Height-Related Polygenic Variants Are Associated with Metabolic Syndrome Risk and Interact with Energy Intake and a Rice-Main Diet to Influence Height in KoGES
Source: Nutrients. 2023 Apr 4;15(7):1764. doi: 10.3390/nu15071764 (PMC10096788; doi:10.3390/nu15071764)
Supplement: Supplementary file 1 [file nutrients-15-01764-s001.zip › nutrients-2314421-supplementary.pdf]

Supplementary Table S1. Generalized multifactor dimensionality reduction (GMDR) results of multi-locus interaction with genes related to adult height

	Model 1				Model 2			
Model 1	TRBA	TEBA	P value	CVC	TRBA	TEBA	P value	CVC
*CPZ*_rs3756173	0.5220	0.5211	0.001	8	0.524	0.5211	0.0107	8
*ZBTB38*_rs6762722 *PAIP2B*_rs13034890	0.5333	0.5334	0.001	9	0.5335	0.5336	0.001	9
*CPZ*_rs3756173 model 2	0.5374	0.5322	0.001	9	0.5374	0.532	0.001	9
*LTBP1*_rs4630744 plus model 3	0.5453	0.5461	0.001	10	0.5453	0.54	0.001	10
*LCORL*_rs7700107, *ADAMTSL3*_rs1600640, *LTBP1*_rs4630744, plus model 2	0.5540	0.5347	0.001	6	0.554	0.5348	0.001	6
*GDF5*_ rs224331, *LCORL*_rs7700107, *DIS3L2*_rs1249260, *LTBP1*_rs4630744 plus model 2	0.5733	0.5244	0.001	8	0.5733	0.5241	0.001	8
*NCAPG*_rs2074974 plus model 6	0.6096	0.5215	0.001	10	0.6096	0.52	0.001	10
*ADAMTSL3*_rs1600640 plus model 7	0.6552	0.5145	0.001	10	0.6554	0.5149	0.001	10
*CPZ*_rs3756173 plus model 8	0.7002	0.5114	10	10	0.7005	0.5111	10	10
*IGF1R*_rs2871865 plus model 9	0.7340	0.5126	0.0107	10	0.7342	0.5122	0.0107	10

TRBA, training balanced accuracy; TEBA, testing balanced accuracy; CVC, cross-validated consistency; P-value for assessing the best model.

Covariates for model 1: sex, area, age, weight, weight at age 18, education, and income. Covariates for model 2: covariates for model 1 plus smoking, energy intake, alcohol intake, physical exercise, and.

Figure S1. Distribution of genetic variants for tall stature by a genome-wide association study.

A. Manhattan plot of the *p*-value of genetic variants for tall stature.

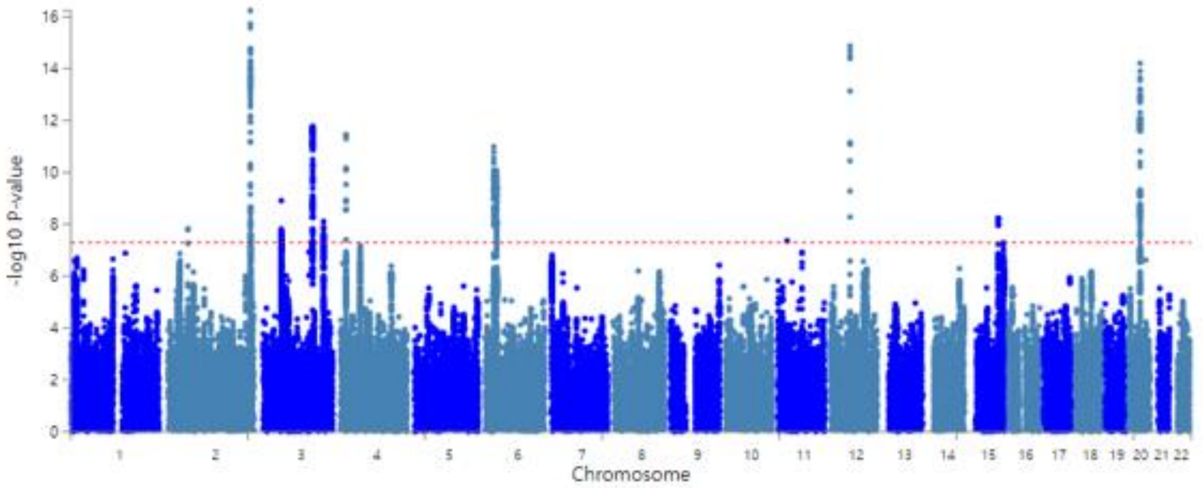

B. Q–Q plot of observed and expected *p*-values for tall stature.

The short stature (SS; n=51,165; control) and tall stature (TS; n=7,536; case) groups of the participants were divided by the cutoff of ≥175 cm for men and ≥163 cm for women, which were the 15th percentiles of adult height in people aged >40 years in 2010-2014 in Korea. The covariates for GWAS were age, gender, weight, residence area, education, income, energy intake, exercise, alcohol drinking, smoking, and osteoporosis incidence.

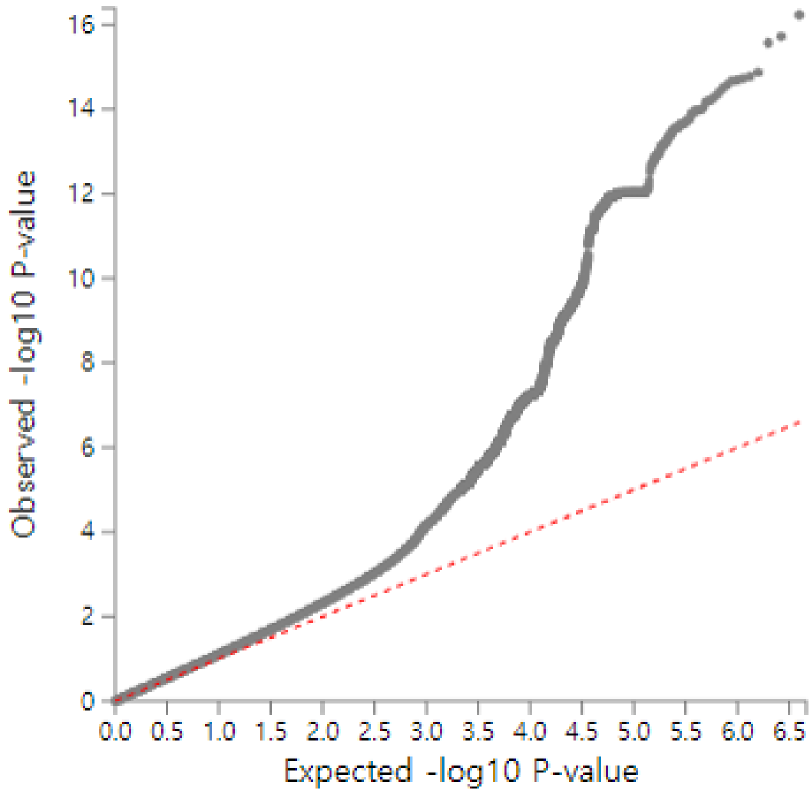

Observed -log10 P-value
Expected -log10 P-value
0
2
4
6
8
10
12
14
16
0.0
0.5
1.0
1.5
2.0
2.5
3.0
3.5
4.0
4.5
5.0
5.5
6.0
6.5
